# Spatio-temporal Genetic Structuring of *Leishmania major* in Tunisia by Microsatellite Analysis

**DOI:** 10.1371/journal.pntd.0004017

**Published:** 2015-08-24

**Authors:** Myriam Harrabi, Jihène Bettaieb, Wissem Ghawar, Amine Toumi, Amor Zaâtour, Rihab Yazidi, Sana Chaâbane, Bilel Chalghaf, Mallorie Hide, Anne-Laure Bañuls, Afif Ben Salah

**Affiliations:** 1 Institut Pasteur, Tunis, Tunisia; 2 Faculté des Sciences de Bizerte-Université de Carthage, Tunis, Tunisia; 3 UMR MIVEGEC (IRD 224-CNRS5290-Universités Montpellier 1 et 2), Centre IRD, Montpellier, France; Charité University Medicine Berlin, GERMANY

## Abstract

In Tunisia, cases of zoonotic cutaneous leishmaniasis caused by *Leishmania major* are increasing and spreading from the south-west to new areas in the center. To improve the current knowledge on *L*. *major* evolution and population dynamics, we performed multi-locus microsatellite typing of human isolates from Tunisian governorates where the disease is endemic (Gafsa, Kairouan and Sidi Bouzid governorates) and collected during two periods: 1991–1992 and 2008–2012. Analysis (F-statistics and Bayesian model-based approach) of the genotyping results of isolates collected in Sidi Bouzid in 1991–1992 and 2008–2012 shows that, over two decades, in the same area, *Leishmania* parasites evolved by generating genetically differentiated populations. The genetic patterns of 2008–2012 isolates from the three governorates indicate that *L*. *major* populations did not spread gradually from the south to the center of Tunisia, according to a geographical gradient, suggesting that human activities might be the source of the disease expansion. The genotype analysis also suggests previous (Bayesian model-based approach) and current (F-statistics) flows of genotypes between governorates and districts. Human activities as well as reservoir dynamics and the effects of environmental changes could explain how the disease progresses. This study provides new insights into the evolution and spread of *L*. *major* in Tunisia that might improve our understanding of the parasite flow between geographically and temporally distinct populations.

## Author Summary

In Tunisia, zoonotic cutaneous leishmaniasis (ZCL) constitutes a significant public health problem. Since 1884, the Gafsa, Kairouan and Sidi Bouzid governorates are the most endemic areas of ZCL. This study used a multi-locus microsatellite typing approach to study the evolution and the population dynamics of *Leishmania major* in Tunisia. Within the same area, in twenty years, parasite populations evolved by producing a genetically differentiated population, probably better adapted to the ecosystem. In agreement with the reported human cases of ZCL, the genetic data on samples from the three governorates shows that the disease did not spread according to a geographical gradient. Furthermore, *L*. *major* flows seem to still occur between governorates and neighboring districts. This study suggests that environmental changes, human activities and reservoir systems have influenced the spread and evolution of *L*. *major* populations. Our findings provide important knowledge on the epidemiology of *L*. *major* in Tunisia and might help understanding why the disease is still spreading from the south to the center, despite the control measures that have been put into place.

## Introduction

**Fig 1:**
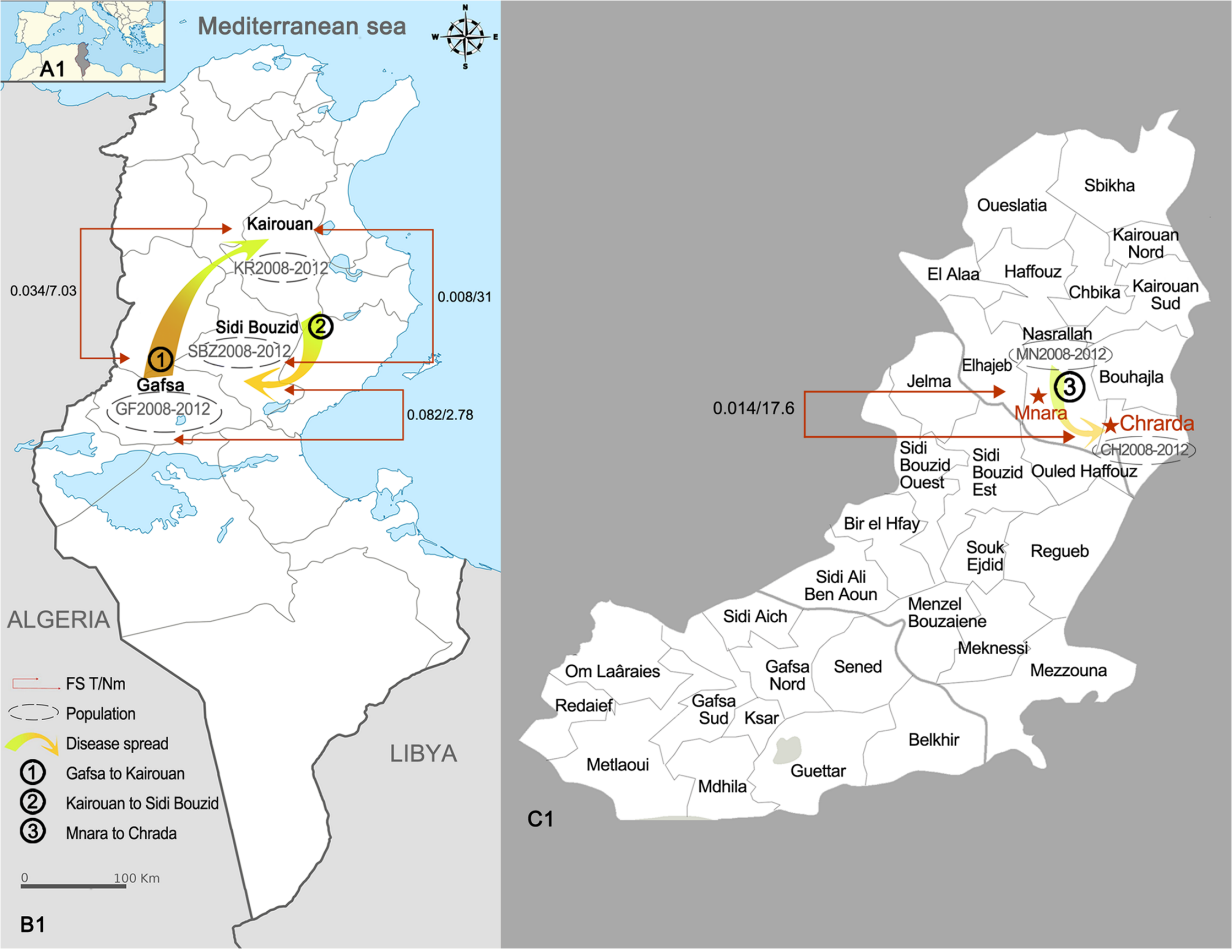
Map of Tunisia and schematic illustration of gene flow between the study areas. (A1) Location of Tunisia, North Africa. (B1) Location of the Gafsa, Sidi Bouzid and Kairouan Governorates within Tunisia. (C1) Zoom of the area under study to show Chrarda and Mnara (Nasrallah delegation) (Mnara and Chrarda are shown in the map with red asterisks) in the Kairouan Governorate.

In Tunisia, zoonotic cutaneous leishmaniasis (ZCL), also known as “Le Bouton de Gafsa” (the pimple of Gafsa), was first described in 1884 by Déperet and Boinet in the Gafsa governorate (south-west of Tunisia) [1]. ZCL represents a typical model of emerging and reemerging zoonosis [2]. ZCL can cause substantial morbidity because of the presence of chronic skin ulcers and the psychological effect of disfigurement [3]. No vaccine is available yet and the current treatments (mainly intra-lesion injections) are expensive and not easy to administer, particularly to children and patients with multiple lesions. For this reason, an international research partnership was launched in 1995 to focus on clinical trials of topical preparations, mainly paromomycin ointments, as new treatments of ZCL caused by *Leishmania major* [4,5,6]. Moreover, epidemiological studies have attempted to determine the spatial and temporal dynamics of ZCL epidemics to improve the prediction of their occurrence and consequently their control [7,8]. ZCL has been endemo-epidemic in the Gafsa region for many years, and in 1982 an epidemic was recorded in the Kairouan governorate for the first time [9]. Then, the disease spread to Sidi Bouzid, where it emerged as an epidemic in 1991 (see map of Tunisia in Fig 1 to localize these regions) [10,11]. Since then, the disease is maintained in these areas and has expanded also to other governorates in the center and south of Tunisia [12].

*L*. *major* is transmitted by the sand fly vector *Phlebotomus papatasi* [13] and rodents are the reservoir, including *Psammomys obesus* (fat sand rat) and *Meriones shawi* (Shaw’s jird) [14,15]. The majority of *L*. *major* strains isolated in Tunisia belong to the MON-25 zymodeme [11,16,17]. It is largely recognized that the population structure of pathogens is influenced by different evolutionary factors, particularly during invasion of new ecosystems [18]. However, it is not known how their geographical distribution and temporal emergence affected the genetic structure and evolution of Tunisian *L*. *major* populations, mainly because multi-locus enzyme electrophoresis (MLEE), which has been widely used for phylogenetic typing of parasites, is not discriminative enough. On the other hand, microsatellite markers in combination with recent statistical methods represent a powerful tool for studying the population structure and monitoring the dynamics of these pathogens in time and space because of their high discriminating power and their presumed neutrality [19,20]. Nevertheless, no detailed study has been carried out on the population structure of *L*. *major* in Tunisia with these powerful markers.

Therefore, the aim of this study was to analyze the spatio-temporal organization of *L*. *major* in the three main endemic areas of ZCL in Tunisia (Gafsa, Kairouan and Sidi Bouzid governorates) by using a multi-locus microsatellite typing approach and population genetic statistical methods. Human isolates collected in the three governorates between 2008 and 2012 were used to assess the population dynamics of *L*. *major* according to the geographical distribution and the chronological emergence of leishmaniasis epidemics in human populations. Moreover, human isolates collected in Sidi Bouzid governorate in the 1991–1992 and 2008–2012 periods were compared to provide information on the evolution of *L*. *major* populations in the same area over twenty years.

## Material and Methods

### Ethics statement

Research included in this study was subject to ethical review by the International Review Board of the Pasteur Institute of Tunis and approved as part of a research project submitted in response to a “call” from the National Institutes of Health for funding Tropical Medicine Research Centers. The Pasteur Institute of Tunis, the study sponsor, took out an insurance policy in accordance with the Tunisian law concerning clinical research. All clinical or biological investigations were performed in the Health Centers of each governorate to guarantee the patients’ safety, confidentiality and respect. All human samples of *Leishmania* were isolated from patients after collection of the written informed consent at the time of the clinical examination. Human isolates were anonymized and the associated information coded for privacy-preserving data mining.

### Geographical origin and collection time of the biological samples

A total of 172 parasite samples, taken from the swollen edge of cutaneous lesions, were collected from patients in different governorates of Tunisia. Isolates from Sidi Bouzid governorate were collected during the 1991–1992 (historical isolates) and the 2008–2012 (recent isolates) periods, whereas isolates from Gafsa and Kairouan were only from the 2008–2012 period (recent isolates) (Table 1). Isolates were stored in the cryobank of the Department of Medical Epidemiology, Pasteur Institute of Tunis. Samples were isolated and typed in the framework of different research projects related to ZCL in Tunisia. Isolates were identified as *L*. *major* by sequence analysis of the gene encoding RNA polymerase II [21] in combination with the MLEE technique [22] at the Centre National de Référence des Leishmanioses (CNRL) of Montpellier, France.

**Table 1:** Geographic sites, year of isolation and number of *L*. *major* isolates included in the study.

Site (governorate)	Recent isolates (2008–2012)	Historical isolates (1991–1992)
Metlaoui (Gafsa)	49	0
M’dhila (Gafsa)	17	0
Chrarda (Kairouan)	31	0
Mnara (Kairouan)	15	0
Oueled Haffouz (Sidi Bouzid)	24	0
Sidi Bouzid Centre (Sidi Bouzid)	0	35
Hania (Sidi Bouzid)	1	0
Total = 172 isolates		

Population dynamics were investigated at the: i) spatial scale, using recent isolates from Gafsa (GF2008-2012) (66 strains), Kairouan (KR2008-2012) (46 strains) and Sidi Bouzid (SBZ2008-2012) (25 strains) and ii) temporal scale, using the recent isolates (SBZ2008-2012, n = 25 samples) and the historical isolates (SBZ1991-1992, n = 35 samples) from the Sidi Bouzid governorate. Furthermore, the recent isolates from the Kairouan governorate (KR2008-2012, 46 strains) were subdivided in two groups based on the history of leishmaniasis infection in the area: 15 isolates from the historical focus of Mnara (MN2008-2012), where the first outbreak occurred in 1982, and 31 isolates from the recent focus of Chrarda (CH2008-2012), where the epidemic started only in 2005 (Fig 1 and Table 1).

### DNA extraction and microsatellite genotyping

For all samples, total genomic DNA was extracted from parasite mass cultures (promastigotes) using DNA extraction kit, according to the manufacturer’s protocol. DNA quality was checked by agarose gel electrophoresis and the concentration measured with a NanoDrop spectrophotometer. Amplification was carried out using fluorescent-labeled primers for ten polymorphic microsatellite markers and the PCR conditions previously described for *L*. *major* [20]. 1μl of PCR product was added to a standard loading mix with 0.5μl of internal lane size standard, and 13.5μl of formamide. Genotyping was performed by capillary electrophoresis using an automatic DNA sequencer. Fragment size was determined automatically using the GeneMapper 4.0 software.

### Microsatellite data analysis

The FSTAT Version 2.9.3.2 software [23] updated from [24] was used to compute estimates and to test the significance of the various population genetic parameters. Genetic polymorphism was measured based on the allelic richness (*A*) and the Nei's unbiased estimate of genetic diversity within subsamples (*H*s) [25]. The observed heterozygosity (*H*o) and expected heterozygosity (*H*e) were also calculated. The Wright's *F* statistics [26] were estimated with the Weir and Cockerham's method [27]: *F*_IS_ measures the relative inbreeding of individuals due to the local non-random union of gametes in each subpopulation, and *F*_ST_ measures the relative inbreeding in subpopulations attributable to the subdivision of the total population into subpopulations of limited size. Therefore, *F*_ST_ also measures the genetic differentiation between subpopulations. The significant departure from 0 of these parameters was tested by 10,000 randomization procedures with FSTAT. The genetic differentiation between historical and recent populations, between governorates (Gafsa, Kairouan and Sidi Bouzid) and between the two sub-populations (historical and recent focus) in the Kairouan governorate was explored. The significance of these estimates was confirmed by *p*-values ≤0.05. The gene flow or migration rate between populations was also estimated using FSTAT, as *Nm = 1- F*_ST_ */4 F*_ST_ [28]. A neighbor-joining tree [29], based on the Cavalli-Sforza and Edward’s chord distances [30], was used to cluster the genotypes. Data were computed using the POPULATION software to build the distance matrix (version 1.2.28; CNRS, UPR9034, Langella, O.) and the tree was generated using FigTree, version 1.4.1 [31].

Finally, data were analyzed using a Bayesian model-based approach implemented in STRUCTURE, version 2.3.4 [32], to explore the structure of the *L*. *major* populations. STRUCTURE uses Bayesian Monte-Carlo Markov Chain (MCMC) sampling to identify the optimal number of clusters K for a given multi-locus dataset, without requiring the identification of the population subunits a priori. The parameters used were the admixture model with the length of burn-in period of 200,000 iterations, followed by 200,000 MCMC repeats after burn-in. Based on the multi-locus genotype data, isolates were divided into K subpopulations with K ranging from 1 to 10 and ten independent runs were performed for each value of K. The K optimal value (i.e., the optimal number of clusters in the dataset) was calculated using STRUCTURE HARVESTER, web version [33]. Two approaches were used to choose *K*. First, ΔK, which measures the second-order rate of change in the log likelihood of the data between successive values of K, was estimated [34]. Second, posterior probabilities for the values of K with the highest Ln P(X|K) were compared. STRUCTURE 2.3.4 was also used to identify migrants. In this case, prior population information was used in the USEPOPINFO option of STRUCTURE. Populations defined according to geographic and temporal criteria (GF2008-2012, KR2008-2012 and SBZ2008-2012, SBZ1991-1992, MN2008-2012 and CH2008-2012) were used as prior population information for this test. Run conditions for this analysis were as mentioned above. As no information was available about migration, a range of migration rates was assigned (MIGPRIOR = 0.01, 0.05, 0.1), as a sensitivity test during the analysis.

## Results

### Analysis of *L*. *major* population structure in the different governorates

The data obtained from the ten polymorphic microsatellite markers [20] were used to assess and compare the genetic variability of isolates collected from patients in the three Tunisian governorates between 2008 and 2012. The Gafsa isolates (GF2008-2012; n = 66) included 48 genotypes (genotypic diversity = 0.73), the Kairouan isolates (KR2008-2012; n = 46) 35 genotypes (genotypic diversity = 0.76) and the Sidi Bouzid isolates (SBZ2008-2012; n = 25) 24 different genotypes (genotypic diversity = 0.96). This finding shows the high *L*. *major* genotypic diversity in these three areas where ZCL is endemic. Unique genotypes and original microsatellite profiles were identified in 18 GF2008-2012 isolates, in four SBZ2008-2012 isolates and in 11 KR2008-2012 samples (S1 Table). Comparisons of the genetic diversity data for the three geographic groups revealed that intraspecific genetic diversity (*H*_s_) was highest in the Gafsa and lowest in the Sidi Bouzid isolates (Table 2). Similarly, the allelic richness (*A*) and the mean observed heterozygosity (*H*_*o*_) decreased progressively from Gafsa to Sidi Bouzid (Table 2).

**Table 2:** Genetic diversity indices, estimated from microsatellite data (10 loci), for the 172 *L*. *major* isolates analyzed in this study.

Population (Area/number of isolates)	Descriptive statistics				
	*A*	*H*s	*H*o	*He*	*F*_IS_
Historical population (Sidi Bouzid/35) SBZ1991-1992	2.4	0.129	0.1311	0.128	0.514
Recent population (Sidi Bouzid /25) SBZ2008-2012	2	0.236	0.056	0.233	0.797
Governorate 1 (Gafsa/66) GF2008-2012	3.2	0.321	0.059	0.319	0.816
Governorate 2 (Kairouan/46) KR2008-2012	2.7	0.306	0.054	0.303	0.823
Governorate 3 (Sidi Bouzid/25) SBZ2008-2012	2.0	0.236	0.048	0.233	0.797
Historical focus (Mnara-Kairouan/15) MN2008-2012	2.2	0.278	0.033	0.271	0.880
Emerging focus (Chrarda-Kairouan/31) CH2008-2012	2.3	0.316	0.064	0.314	0.796
Whole sample (172)					

*L*. *major* isolates were included in the different subpopulations: historical (SBZ1991-1992) and recent populations (SBZ2008-2012) from the Sidi Bouzid governorate; recent populations from Gafsa (GF2008-2012), Kairouan (KR2008-2012) and Sidi Bouzid (SBZ2008-2012); the (KR2008-2012) population was further divided in samples from Mnara (MN2008-2012, historical epidemic focus) and from Chrarda (CH2008-2012, emerging epidemic focus).

*A* = allelic richness per population based on the standardized minimal sample size; *H*s = gene diversity; *Ho* = observed heterozygosity; *He* = expected heterozygosity; *F*_IS_ = inbreeding coefficient.

The mean expected heterozygosity (*H*_e_) values were much higher than the *H*_o_ values in all three populations (Table 2). These results were confirmed by the inbreeding coefficient (*F*_IS_) values estimated for each locus and in each population, revealing a deficit in heterozygosity in all three populations (Table 2). The *F*_*ST*_ values, which are used as a measure of genetic differentiation between populations, were very low, but significantly different between the GF2008-2012 and SBZ2008-2012 isolates and between the GF2008-2012 and KR2008-2012 isolates. Conversely, the *F*_*ST*_ values were not significantly different between the KR2008-2012 and SBZ2008-2012 populations (Table 3). Accordingly, the highest migration rate (*Nm*) value was between the KR2008-2012 and SBZ2008-2012 populations, whereas the *Nm* values were much lower between the GF2008-2012 and KR2008-2012 and between the GF2008-2012 and SBZ2008-2012 populations (Table 3).

**Table 3:** Differentiation measures (*F*_ST_), probabilities (*P*-*value*) and migration rate (*N*_m_) between subpopulations.

Subpopulation (Area) (Number of isolates)	*F*_ST_	*P-value* *	*Nm (migrant/population)*
Historical (Sidi Bouzid) (35) versus Recent (Sidi Bouzid) (25)	0.213	0.05	0.923
Governorate 1 (Gafsa) (66) versus Governorate 2 (Kairouan) (46)	0.034	0.05	7.03
Governorate 1(Gafsa) (66) versus Governorate 3 (Sidi Bouzid) (25)	0.082	0.016	2.78
Governorate 2 (Kairouan) (46) versus Governorate 3 (Sidi Bouzid) (25)	0.008	0.083	31
Emerging focus (Chrarda-Kairouan) (31) versus Historical focus (Mnara-Kairouan) (17)	0.014	0.05	17.60

*Data were considered significant when *P*-value ≤ 0.05.

**Fig 2:**
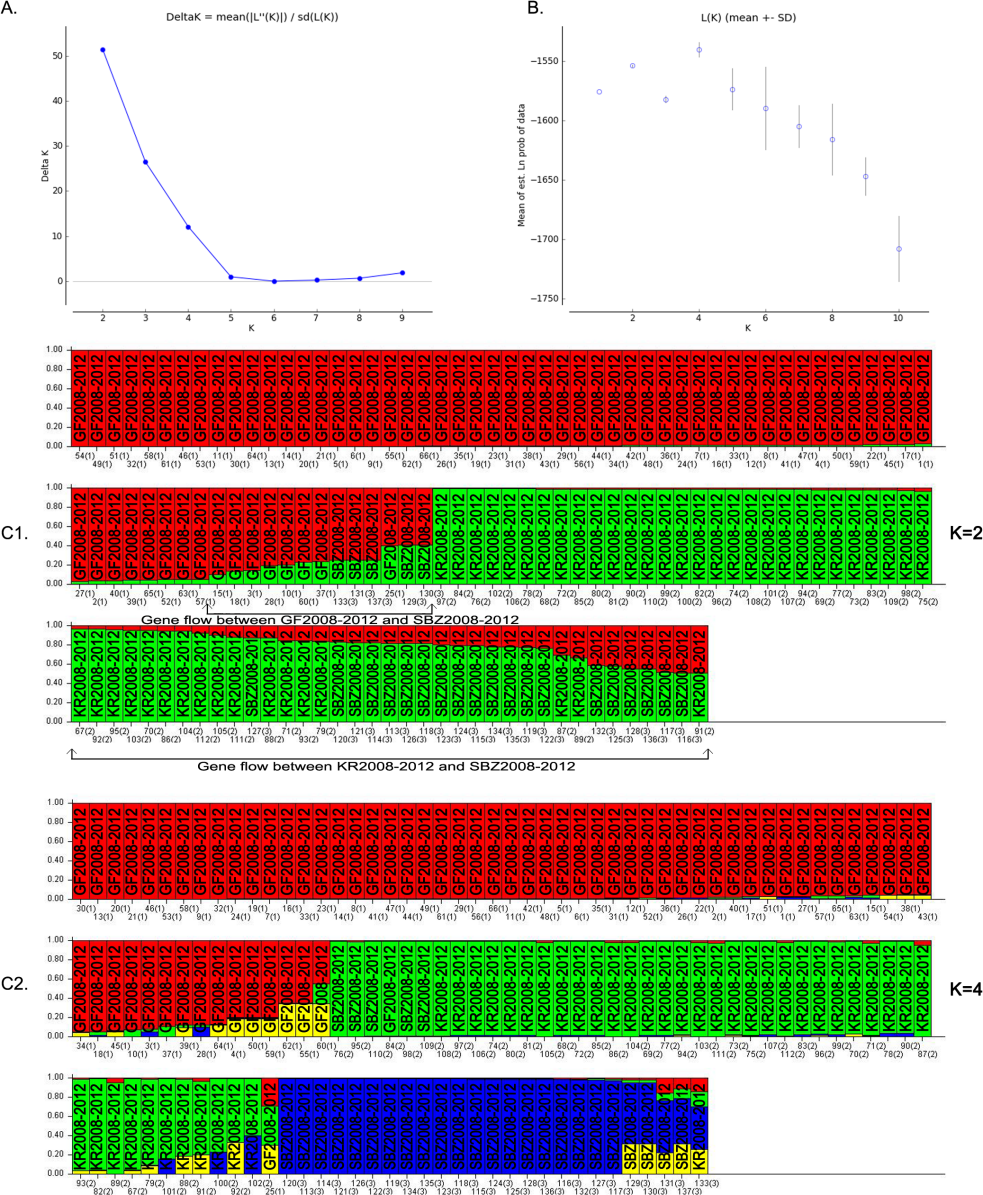
Estimated population structure of *L*. *major* in Tunisia as inferred by the STRUCTURE software on the basis of the data on 10 microsatellite markers obtained for 137 recent isolates from the Gafsa (GF2008-2012; n = 66), Kairouan (KR2008-2012; n = 46) and Sidi Bouzid (SBZ2008-2012; n = 25) governorates. (A) Plot of the mean posterior probability (LnP(D)) values per clusters (K), based on 10 replicates per K, generated by the STRUCTURE software, and (B) delta-K analysis of LnP(K). (C) STRUCTURE plots grouped by Q-matrix (estimated membership coefficient for each sample) showing the distribution of genetic variation (C.1) at K = 2 and (C.2) at K = 4. Each strain is represented by a vertical line, which is partitioned into the colored segments that represent the parasite estimated membership fractions in K. The same color indicates that the isolates belong to the same group. Different colors for the same isolate indicate the percentage of the genotype shared with each group. Gene flow between populations is indicated with arrows.

The Bayesian model-based clustering analysis implemented in STRUCTURE indicated that our dataset (GF2008-2012, KR2008-2012 and SBZ2008-2012 isolates) could be organized in four (maximum L(K)) or two (maximum ΔK) clusters (K) (Fig 2A and 2B). For K = 2, 92% of *L*. *major* isolates from Gafsa, 10% from Kairouan and 38% from Sidi Bouzid were included in one of the inferred clusters and the remaining samples in the second one (Fig 2C.1). Based on the *Q*-matrix bar plots obtained for each isolate by calculating the posterior probabilities of belonging to each K cluster, the SBZ2008-2012 population showed a mixed membership to the inferred clusters (Fig 2C.1). For K = 4, the *Q*-matrix bar plots showed an increased separation of the *L*. *major* sample substructure. Although the most likely number of groups here suggested a total of four populations no strains were fully assigned to the fourth putative group (yellow group) (Fig 2C.2), suggesting that this was not a valid population for this set [34,35]. This “phantom” population suggesting a wider and deeper clinical sample collection may discover new diversity even in this small geographic area [36]. Based on the bar plots for the two assumptions, K = 2 seems to be the most probable partition for our data set. In agreement with the very low genetic differentiation between governorates, the analysis carried out with the STRUCTURE program did not divide the three populations according to their geographical origin. Nevertheless, most isolates from Gafsa and from Kairouan were grouped in cluster 1 and cluster 2, respectively, and the samples from Sidi Bouzid were distributed in the two clusters (38% in cluster 1 and 62% in cluster 2). Strains showing mixed membership were observed in each population, probably due to the low level of differentiation among populations. The occurrence of gene flow between GF2008-2012 and SBZ2008-2012, as well as between SBZ2008-2012 and KR2008-2012 can be clearly observed in Fig 2C.1. In the STRUCTURE assignment tests, we only reported the results for MIGPRIOR = 0.1, because migration appeared to occur frequently between governorates, thus the optimal MIGPRIOR value was likely to be the highest one [32]. Furthermore, when running the migration model at K = 3 (equal to the number of predefined populations), ten samples were identified as migrants. Among these migrants, two GF2008-2012 samples were assigned to the KR2008-2012 population and one GF2008-2012 sample to the SBZ2008-2012 group. Among the KR2008-2012 samples, only two showed a posterior probability of having recently migrated from Sidi Bouzid. In the SBZ2008-2012 group, five samples were assigned to both Gafsa and Kairouan. These results show a recent connectivity between localities. According to the *Q*-values of the samples, the KR2008-2012 and SBZ2008-2012 populations seemed to be more inter-connected than the GF2008-2012 population with either SBZ2008-2012 or KR2008-2012.

The low genetic differentiation (F-statistical approach), the recent migration events (Bayesian analysis), the gene flow over the years (F-statistical approach) and the absence of clear separation (Bayesian analysis) between governorates were also confirmed by the finding that the phenetic tree did not highlight any subdivision of the strains according to their geographical origin. (S1 Fig).

### Analysis of the *L*. *major* population structure in historical and recent isolates

The data of the ten microsatellite markers were used also to compare the 35 historical (SBZ1991-1992) and 25 recent (SBZ2008-2012) isolates from Sidi Bouzid governorate. Two of the microsatellites were monomorphic in the SBZ1991-1992 population, revealing 26 genotypes in the 35 isolates (0.74). Analysis of the SBZ2008-2012 isolates revealed three polymorphic microsatellites that generated 24 genotypes (0.96). The number of alleles per locus ranged from 1 to 8, with an allelic richness (*A*) of 2.4 in the historical population and of 2 in the recent isolates (S1 Table). The pattern of unbiased gene diversity (*H*s) increased over time (Table 2). Comparison of the mean *H*_e_ and *H*_o_ values showed a departure from the expected values in SBZ2008-2012 population, whereas both observed and expected heterozygosity were highly similar for SBZ1991-1992 population and no departure from expected values was detected for these strains (Table 2). Indeed, as reported in previous studies, *H*_o_ was much lower than *H*_e_ and the inbreeding coefficient *F*_IS_ revealed a strong heterozygote deficiency in both populations (Table 2). The *F*_ST_ value (*F*_ST_ = 0.213) showed an important genetic differentiation between historical and recent isolates and the migration rate *Nm* was very low (0.923) (Table 3).

**Fig 3:**
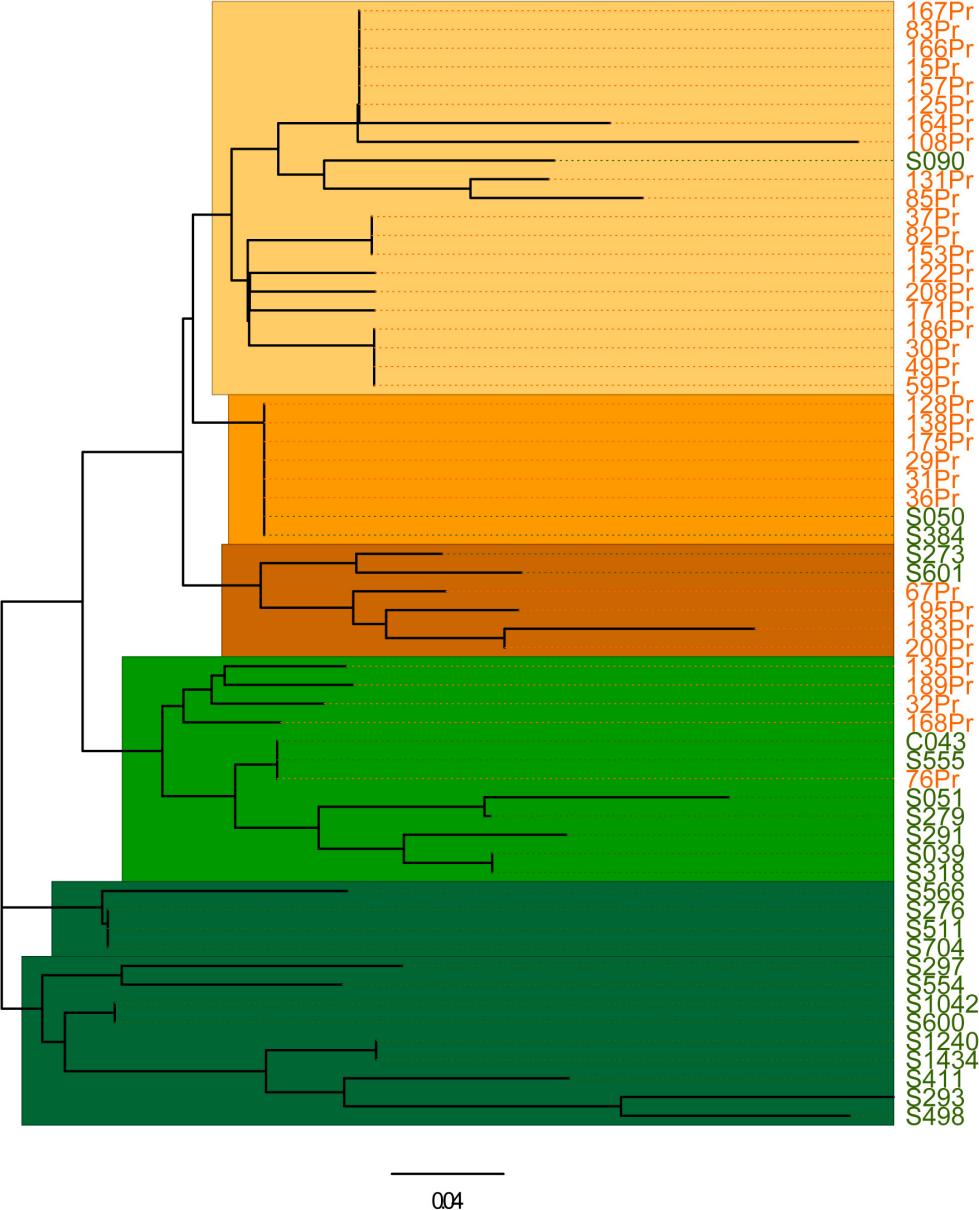
The unrooted neighbor-joining tree inferred from pair-wise Cavalli-Sforza and Edwards’ chord distances based on the 10 microsatellite data of 60 *Leishmania* major isolates (SBZ1991-1992 and SBZ2008-2012) shows that the historical isolates (SBZ1991-1992, n = 35; orange labels) and the recent isolates (SBZ2008-2012, n = 25; green labels) can be subdivided in five clusters.

The Bayesian model-based clustering analysis indicated two clusters as the most probable genetic structure of these two populations. The first cluster included most of the historical samples (98.8%), which shared a common genetic background based on the genotyping results, and 19.3% of the recent isolates. The second cluster was mainly (80.7%) composed by recent isolates. STRUCTURE identified 0.42 and 4.82 misclassified samples for the historical and recent populations, respectively. However, as the migration model used different prior and modeling assumptions for identifying migrants, three SBZ2008-2012 isolates showed contrasting patterns of assignment, suggesting high genetic connectivity over time. The Neighbor Joining tree revealed no strict partition between the historical and recent populations (see Fig 3). The tree included five main clusters that corresponded to the two populations obtained with the STRUCTURE analysis: the two upper clusters matched the first STRUCTURE population, while the three remaining clusters corresponded to the second STRUCTURE population.

### Analysis of the *L*. *major* population structure relative to the two ZCL emergence periods in Kairouan

The recent isolates from the Kairouan governorate (KR2008-2012, n = 46) came from the historical focus (1982) of Mnara (MN2008-2012, n = 15) and from the emerging focus (2005) of Chrarda (CH2008-2012, n = 31). Analysis of the genetic data of these two sub-populations showed that six of the ten tested microsatellite loci were polymorphic (4GTG, 39GTG, 45GTG, 1GC, 71AT and 1GACA) in both populations. The remaining four markers (27GTG, 36GTG, 28AT and 1CA) were monomorphic in one or the other population (S1 Table). The allelic richness (*A)* ranged from 2.2 (MN2008-2012 isolates) to 2.3 (CH2008-2012 isolates) (Table 2). *H*_s_, *H*_o_, *H*_e_ and *F*_IS_ were higher in the CH2008-2012 (emerging focus) than in the MN2008-2012 population. As in all the populations assessed in this study, the mean *H*_o_ values in the two sub-populations were extremely low compared to the *H*_e_ values. The heterozygosity deficit (measured by *F*_IS_) was very high in both populations (Table 2).

The *F*_ST_ value showed that the genetic divergence in the two foci was very low, but significant (Table 3), indicating a genotype flow between populations. Quantification of the migration events in the two districts using F-statistical approaches, revealed 17 migrants per population. The Neighbor joining analysis confirmed that there was no genetic structuring between populations (S2 Fig). The posterior probabilities of belonging to a K cluster and the corresponding ΔK values calculated with the STRUCTURE software showed a peak at K = 2, indicating that two genetic clusters were the best solution for this dataset. The STRUCTURE analysis did not detect any recent migrant over the last generations between the partitions defined by the software. The different migration rates observed with the two methods can be explained by the fact that STRUCTURE only identifies very recent migrants, whereas the F-method may highlight migration events that occurred hundreds or thousands of years ago (Pritchard, personal communication).

## Discussion

The Sidi Bouzid, Gafsa and Kairouan governorates are the areas where ZCL is most endemic in Tunisia. Despite the cyclic occurrence of outbreaks [8], the annual reported incidence was > 500 cases in Kairouan, and > 1000 cases in Gafsa and Sidi Bouzid in 2004 [37]. In this study, we carried out the first genetic substructure analysis of *L*. *major* populations in Tunisia based on genotype data to investigate the spatio-temporal dynamics and the evolution of this species in these three governorates where most human cases are recorded.

### Spatial organization of *L*. *major* genetic diversity in Tunisia

Our findings revealed the presence of a decreasing genetic gradient from Gafsa to Kairouan and Sidi Bouzid between 2008 and 2012. According to the chronology of leishmaniasis emergence in Tunisia, Gafsa is the oldest and the most well-established focus of ZCL. The first outbreak was described in 1884 in El Guettar, in the southern part of the Gafsa governorate. Conversely, the first cases in the Kairouan and in Sidi Bouzid governorates were recorded only in 1982 and 1991, respectively. However, we do not know whether the chronology of disease emergence corresponds to the real spread of leishmaniasis. Furthermore, the governorate disease reports do not record the exact geographic origin of the infection.

Despite these limitations, this study shows the presence of some genetic differentiation between Gafsa and Sidi Bouzid and Gafsa and Kairouan, but not between Kairouan and Sidi Bouzid. Furthermore, we obtained a gradient of diversity that progressively decreases from Gafsa to Kairouan and then to Sidi Bouzid. These results are in agreement with the chronology of *L*. *major* outbreaks in these three governorates (see above) and do not follow a south-north geographical gradient. It is worth noting that this genetic pattern (i.e., population structuring according to the geography and to the ancientness of the focus) is classically observed for *Leishmania* species mainly because of the low dispersion capacity of sandflies and reservoirs [38]. Moreover, the STRUCTURE analysis could not cluster the three groups of recent isolates according to their provenance and showed more connectivity between Kairouan and Sidi Bouzid than between Gafsa and Sidi Bouzid. The analyses of the genotypic data, using the Bayesian model-based method and F-statistics, suggest that there had been and still there is a flow of genotypes between governorates, especially between Kairouan and Sidi Bouzid. According to the data published by Chargui et al. [18], *L*. *infantum* and *L*. *major* in Tunisia do not seem to follow the same genetic and evolutionary pattern. Indeed, although *L*. *infantum* seems to have spread from the north to the center of the country [16,39], more genetic diversity, particularly high heterozygosity, was found in the center than in the north.

Two hypotheses might explain the genetic differentiation and diversity patterns: 1) leishmaniasis spread first from Gafsa to Kairouan through human activities (economic activities or social development projects) and then from Kairouan to Sidi Bouzid; 2) parasites have evolved in different ways because of different ecosystems. Concerning the second hypothesis, rodent and sandfly ecology could influence the parasite transmission. Indeed, entomologic studies have demonstrated that in Tunisia sandfly populations vary in density and species composition according to the ecological conditions [40]. Furthermore, rodent populations (*P*. *obesus* and *M*. *shawi*) also show a different distribution in the different governorates linked to the food availability [11,41,42]. It is worth noting that, like for other *Leishmania* species (i.e., *L*. *guyanensis*, *L*. *braziliensis* and *L*. *donovani*), we found a strong deficit of heterozygosity in all our populations, in agreement with a recently described mixed-mating system of reproduction (clonality, endogamy, allogamy) [43,44,45,46,47]. Concerning *L*. *infantum* in Tunisia, high level of heterozygosity was observed in Kairouan with evidences of hybridization events [18]. These authors also found a deficit of heterozygosity, although they analyzed the MON-24 and MON-1 populations separately. The different genetic patterns and especially the higher heterozygosity observed in *L*. *infantum* compared to *L*. *major* could be explained by different proportions of the three reproduction mode of *Leishmania* parasites [43,44,45,46,47]. Nevertheless, the sample size in the work by Chargui et al. [18] was too small (27 strains) to do more extensive comparisons.

### Temporal organization of *L*. *major* genetic diversity in Tunisia

First, we explored the evolution of the *L*. *major* population in isolates from Sidi Bouzid governorate between 1991–1992 and 2008–2012. The results show that the recent population is more diverse than the historical one with a significant genetic differentiation over time. In twenty years, *L*. *major* evolved with a change in allelic frequencies. The increase of genetic diversity reflects the accumulation of genetic changes overtime in this population. The continuous presence of some genotypes over the two decades and the grouping in the same cluster of both the recent and historical populations (Fig 2) strongly suggest that the recent population evolved from the historical one. This hypothesis is supported by the detection of migrants between the old and recent population. As ZCL incidence and *Leishmania* genetic diversity have been continuously increasing, we can assume that the *Leishmania* population has gradually adapted to the environment [7,8,48]. Furthermore, in the last twenty years, Tunisia, like the rest of the world, went through rapid ecosystem modifications. For example, the water project (construction of the Sid Saâd dam in the Nasrallah delegation, Kairouan governorate, in 1982) [49], the pest control program (destruction of rodent borrows and elimination of chenopods) and the development of agricultural projects around the city of Sidi Bouzid (Sidi Bouzid governorate, 1992) could have had an effect on temperature, humidity, soil and vegetation. These anthropic modifications might have disturbed the sandfly and rodent populations and thus impacted the evolution of the *Leishmania* population since the 1990’s. Nevertheless, migration events from other regions cannot be excluded. The calculation of genetic differentiation between *L*. *major* population from Sidi Bouzid and those from North Africa [20], Central Asia [20], Middle East [20], Iran [19] and Pakistan [50] revealed considerable genetic differentiation (>0.55) with highly significant *p*-values (≤0.05) (S2 Table).

To further understand the *L*. *major* temporal/spatial dynamics in Tunisia, we also analyzed recent isolates (2008–2012) from two districts within the Kairouan governorate: Mnara (historical focus) and Chrarda (emerging focus). Considering the long interval (23 years) between the human outbreaks in the two districts, we expected that the more recent focus would show a lower genetic diversity than the historical focus. However, the genetic data showed a slightly higher genetic diversity in the more recent focus (Chrarda), despite the low sample size. In parallel, the low differentiation suggests that these are not isolated populations. Indeed, the F-statistics and Bayesian methods estimated that the Mnara and Chrarda isolates are closely related, although the difference was sufficient to correctly assign most samples to their respective district. The migration analysis using both methods suggests the existence of historical migration events (F-statistics method), but not recent migration events (Bayesian method). These results support the hypothesis that the outbreak in Chrarda is the result of the spread of a population, rather than of a small set of genotypes, from Mnara several years ago. Based on the short distance between the Mnara and Chrarda districts (12 to 20 kilometers), the *Leishmania* population spread could be explained by human activities and also by the vector or reservoir dynamics. As sandflies are known to be bad flyers, *Meriones shawi* movements (the disease reservoir) and the human economic and social exchanges could be the main sources of the emergence of *L*. *major* in Chrarda.

### Conclusion

This study brings new insights into the spatial and temporal evolution of *L*. *major* in Tunisia. Over two decades, the *L*. *major* population evolved into a new, genetically differentiated population, probably better adapted to the environment. This could explain the increase of parasite transmission to humans and the higher incidence of ZCL in these areas over the last years [7,8,48].

To control the emergence of *L*. *major* in new areas in Tunisia, it is now essential to identify the routes of spread. Our findings suggest that the parasite population dynamics do not follow a vertical south-north gradient. Indeed, the disease seems to have spread from Gafsa to Kairouan and then to Sidi Bouzid. Human activities and/or the disease reservoir dynamics might explain this geographically non-gradual spread.

When a disease settles in a new area, it normally sources a subset of the original pathogen population and, as a consequence, genetic diversity should be reduced in the new population. Analysis of the Mnara and Chrarda isolates indicates that the two populations are similar with very low differentiation and historical migration events. This genetic similarity suggests the occurrence of high flow of genotypes between these neighboring populations that would be at the origin of the outbreak in Mnara. The relationships between environmental changes, human activities and reservoir systems have doubtlessly influenced the spread and the evolution of the Tunisian *L*. *major* populations as it is largely demonstrated for *Leishmania* species [2]. More work is needed to assess the influence of the movements and population structures of the rodent reservoirs and vectors on *L*. *major* evolution.

## Supporting Information

S1 Fig The unrooted neighbor-joining tree inferred from pair-wise Cavalli-Sforza and Edwards’ chord distances computed from microsatellite data of 137 *Leishmania major* isolates (GF2008-2012, n = 66; green labels, KR2008-2012, n = 46; blue labels and SBZ2008-2012, n = 25, pink labels) does not show any subdivision of the strains according to their geographical origin. (PDF)

S2 Fig The Unrooted neighbor-joining tree inferred from genetic distances (Cavalli-Sforza and Edwards, 1967) computed from microsatellite data of 46 *L*. *major* strains (CH2008-2012 and MN2008-2012) shows no genetic structuring between the historical focus (MN2008-2012, n = 15; blue labels) and the emerging focus (CH2008-2012, n = 31; black labels). (PDF)

S1 Table Sample code, origin and multilocus microsatellite genotyping results of the *L*. *major* isolates analyzed in this study. The microsatellite profiles are designed by three-digit numbers separated by a fraction bar. Each three-digit number assigns the detected allele, coded by the microsatellite size in base pairs (only one size value for homozygous loci and two size values for heterozygous loci).
(XLSX)

S2 Table Differentiation measures (*F*_*ST*_) between Tunisian *L*. *major* populations (historical and recent populations) and *L*. *major* isolates from Central Asia, Africa, Middle East, Iran and Pakistan. (XLSX)
