# Supplementary figures and images for: Spatio-temporal Genetic Structuring of Leishmania major in Tunisia by Microsatellite Analysis
Source: PLoS Negl Trop Dis. 2015 Aug 24;9(8):e0004017. doi: 10.1371/journal.pntd.0004017 (PMC4547700; doi:10.1371/journal.pntd.0004017)

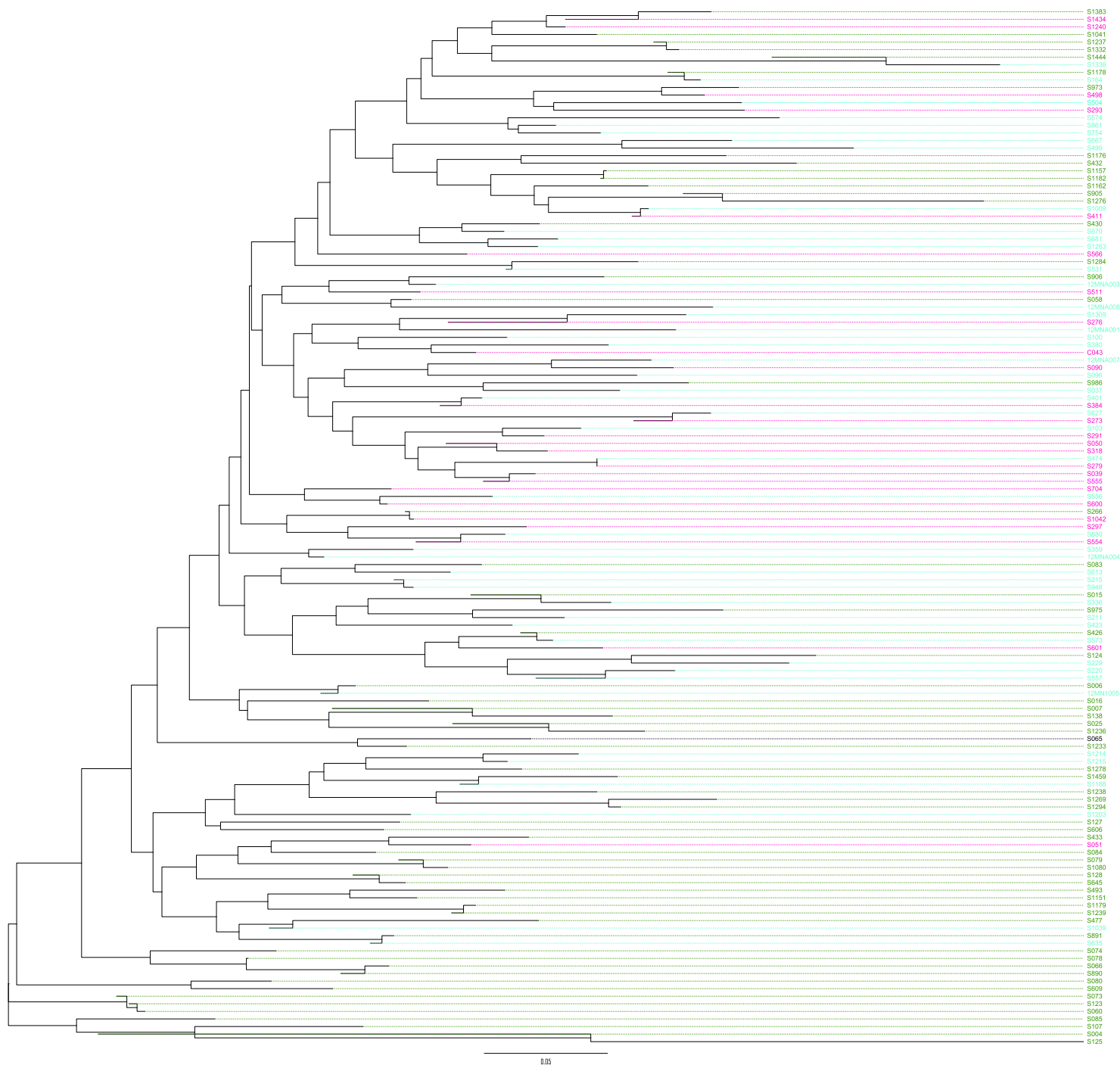

Supplement: S1 Fig — (PDF) [file pntd.0004017.s001.pdf]

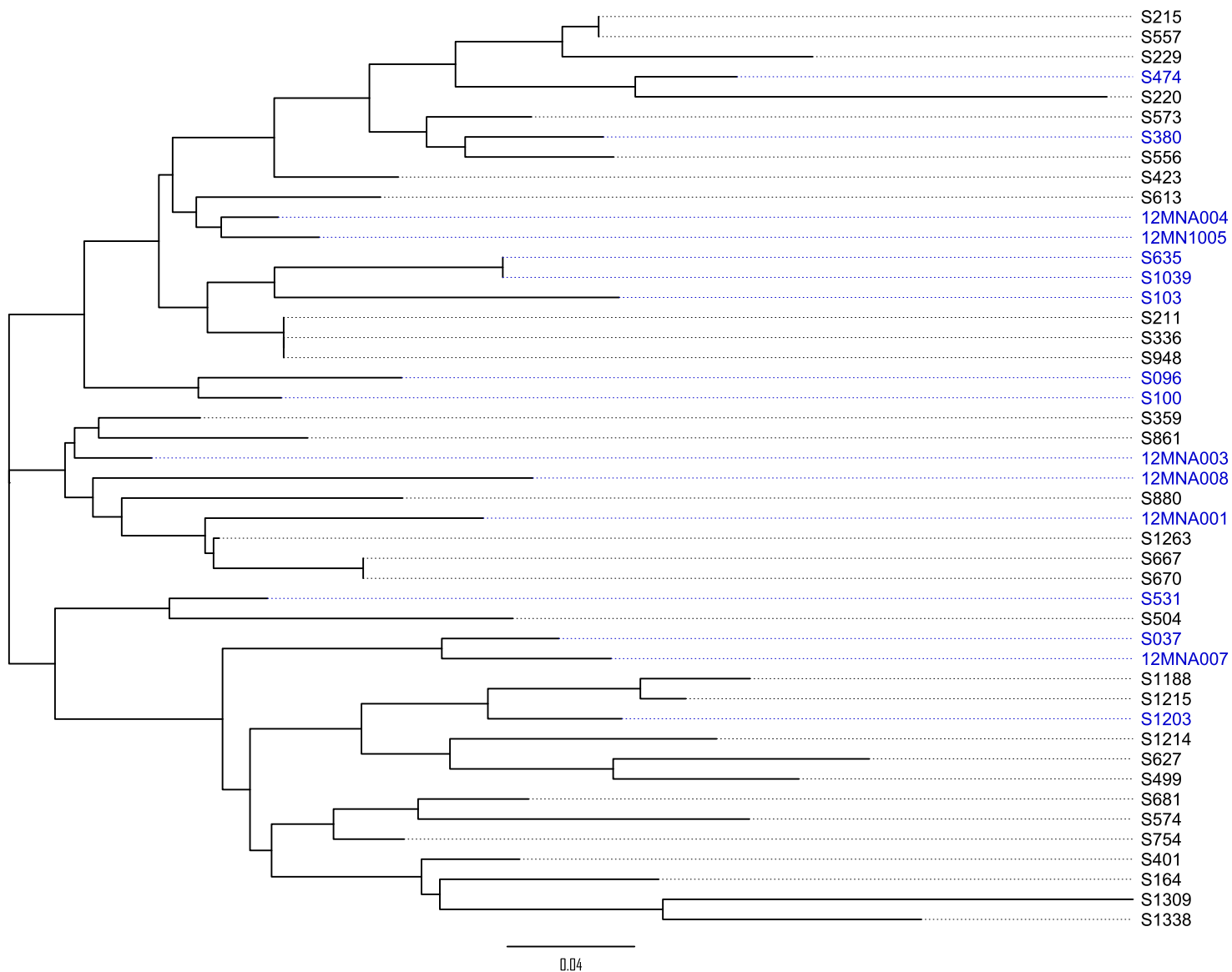

Supplement: S2 Fig — (PDF) [file pntd.0004017.s002.pdf]
